# Myeloid-Derived Suppressor Cells in Cancer and COVID-19 as Associated with Oxidative Stress

**DOI:** 10.3390/vaccines11020218

**Published:** 2023-01-19

**Authors:** Celia María Curieses Andrés, José Manuel Pérez de la Lastra, Celia Andrés Juan, Francisco J. Plou, Eduardo Pérez-Lebeña

**Affiliations:** 1 Hospital Clínico Universitario of Valladolid, Avenida de Ramón y Cajal 3, 47003 Valladolid, Spain; 2 Cinquima Institute and Department of Organic Chemistry, Faculty of Sciences, Valladolid University, Paseo de Belén 7, 47011 Valladolid, Spain; 3 Institute of Natural Products and Agrobiology, CSIC-Spanish Research Council, Avda. Astrofísico Fco. Sánchez, 3, 38206 La Laguna, Spain; 4 Institute of Catalysis and Petrochemistry, CSIC-Spanish Research Council, 28049 Madrid, Spain; 5 Sistemas de Biotecnología y Recursos Naturales, 47625 Valladolid, Spain

**Keywords:** myeloid-derived suppressor cells, cancer, COVID-19, reactive species, ROS, RNS and RHS, innate immunity, antimicrobial

## Abstract

Myeloid-derived suppressor cells MDSCs are a heterogeneous population of cells that expand beyond their physiological regulation during pathologies such as cancer, inflammation, bacterial, and viral infections. Their key feature is their remarkable ability to suppress T cell and natural killer NK cell responses. Certain risk factors for severe COVID-19 disease, such as obesity and diabetes, are associated with oxidative stress. The resulting inflammation and oxidative stress can negatively impact the host. Similarly, cancer cells exhibit a sustained increase in intrinsic ROS generation that maintains the oncogenic phenotype and drives tumor progression. By disrupting endoplasmic reticulum calcium channels, intracellular ROS accumulation can disrupt protein folding and ultimately lead to proteostasis failure. In cancer and COVID-19, MDSCs consist of the same two subtypes (PMN-MSDC and M-MDSC). While the main role of polymorphonuclear MDSCs is to dampen the response of T cells and NK killer cells, they also produce reactive oxygen species ROS and reactive nitrogen species RNS. We here review the origin of MDSCs, their expansion mechanisms, and their suppressive functions in the context of cancer and COVID-19 associated with the presence of superoxide anion ^•^O_2_^−^ and reactive oxygen species ROS.

## 1. Introduction

Myeloid-derived suppressor cells MDSCs are formed from bone marrow progenitor cells when myelopoietic processes are disrupted by various diseases. They proliferate under pathological conditions due to altered hematopoiesis. They differ from other myeloid cell types in that they exhibit immunosuppressive activity, as opposed to immunostimulatory properties, and interact with and even regulate the functions of other immune cells, such as T cells, dendritic cells, macrophages, and natural killer cells NK.

These MDSCs regulate the immune system and related responses associated with various diseases. Excessive production of these MDSCs is considered an important factor in the success or failure of cancer immunotherapy. Elevated levels of MDSCs in the tumor microenvironment TME correlate with poorer survival in patients with solid tumors and may mediate resistance to checkpoint inhibitor therapy CIT. Although their main function is to suppress the response of T cells and NK killer cells, polymorphonuclear MDSCs can generate reactive oxygen species ROS and reactive nitrogen species RNS. The accumulation of excessive intracellular ROS can cause stress to protein folding by altering endoplasmic reticulum calcium channels, thereby disrupting cell homeostasis. One of the main mechanisms by which MDSCs induce immune suppression is mediated by reactive oxygen species ROS. Increased ROS release in MDSCs is caused by augmented activity of NADPH oxidase NOX2 [1]. In this article, we review the function of MDSCs in cancer and COVID-19 disease.

## 2. Myeloid-Derived Suppressor Cells MDSC and Its Role in Immune System

Under physiological conditions, myeloid progenitor cells differentiate into macrophages M, dendritic cells DC, or granulocytes G [2]. MDSCs are formed from bone marrow precursors when myelopoietic processes are disrupted, which occur when various diseases are triggered [3]. Under certain pathological conditions, such as in cancer or infection, myelopoiesis (defined as the production of the bone marrow and the resulting cells: eosinophilic granulocytes, basophilic granulocytes, neutrophilic granulocytes, and monocytes) is abnormal, allowing the accumulation and proliferation of immature myeloid cells that have potent immunosuppressive capabilities [4,5,6,7]. MDSCs were described more than 30 years ago in cancer patients [8]. Common features of MDSCs are their myeloid origin, their immature state and a remarkable ability to suppress T-cell and NK-cell responses [9]. MDSCs are elevated in virtually all patients with cancer and malignancies, and include two main subpopulations of cells: monocytic M-MDSC and granulocytic (polymorphonuclear PMN-MDSC), defined by their expression of plasma membrane markers and their content of immunosuppressive molecules [10]. MDSCs are pathologically activated neutrophils and monocytes and have potent immunosuppressive activity, regulating immune responses in many pathological conditions (including cancer, chronic infection, sepsis, and autoimmunity) and are closely associated with poor prognosis in cancer. MDSCs are a major obstacle to immunotherapies, as accumulation of MDSC populations in circulating leukocytes and tumor infiltrates has been observed in patients who do not respond to checkpoint inhibitor therapy [11,12].

In addition to their suppressive effects on adaptive immune responses, MDSCs regulate innate immune responses by modulating macrophage cytokine production [13]. Non-immunological functions of MDSCs, such as the promotion of tumor angiogenesis and metastasis, have also been described [14]. In pregnancy and neonates, the functions of MDSCs have been described under physiological conditions [15].

MDSCs are multifaceted and use multiple mechanisms to inhibit both adaptive and innate immunity; for example, in the adaptive system, T cells are a primary target. Initial studies showed that MDSCs produce some of their suppressive effects by releasing soluble mediators [16], requiring cell contact due to the short half-life and distribution of the effector molecules. On the one hand, infiltrating T cells are reduced in the tumor microenvironment [17], and at the same time MDSCs limit the migration of T cells to lymph nodes where they could be activated. Recent studies have shown that suppressive activity is also mediated by MDSC-derived exosomes [18].

Activation of MDSCs is mediated by the expression of inflammatory cytokines, such as GM-CSF, IL-6, G-CSF, IL-1β, PGE2, TNF-α, and VEGF and by transcriptional regulators including STAT3, CEBP/β, STAT5, IRF8, S100A8/9, RB, TIPE2, and GCN2 [4,5,6,19]. Growing tumors produce cytokines and other substances that affect the development of MDSC, such as colony-stimulating factors G-CSF and GM-CSF and the MDSC-promoting interleukin IL-6 [20].

Recent results in both tumor mice and cancer patients suggest that increased metabolism of ARG1 by MDSCs inhibits T-cell responses [21]. MDSCs prevent T-cell activation by limiting the availability of amino acids necessary for T-cell proliferation, such as arginine, or by producing substances that block antigen recognition. MDSCs produce arginase 1 ARG1, which competes for the substrate arginine, depleting it, resulting in the loss of the T-cell receptor chain essential for T-cell activation. The main targets of MDSCs are T cells and the main factors involved in immune suppression include ARG1 arginase, iNOS, TGF-β, IL-10, COX2, cysteine sequestration by indoleamine 2,3-dioxygenase IDO, decreased L-selectin expression by T cells, and several others. M-MDSCs and PMN-MDSCs use different immune suppression mechanisms, the former M-type suppress T-cell responses both specifically and non-specifically using mechanisms associated with ^•^NO and cytokine production [22]. PMN-MDSCs can suppress immune responses primarily in an antigen-specific manner and ROS production is essential to maintain this ability [23]. Extravasation of T cells from the blood and lymphatics to the lymph nodes requires the expression of L-selectin/CD62L on T cells. MDSCs express the enzyme ADAM-17, which cleaves L-selectin on T cells, thus preventing extravasation and limiting T-cell entry into lymph nodes [24].

NK-cell cytotoxicity is also inhibited by MDSC [25]. A novel subset of MDSCs specifically targeting NK cells is accumulated in the tumor microenvironment of mice by the proinflammatory cytokine IL-1. Upon their activation by prostaglandin E2 PGE2, MDSCs reduce NK-cell activity in melanoma patients by producing the immunosuppressive transforming growth factor, TGF-1β [26].

Dendritic cells DC are negatively affected by MDSC in a similar way [27], by the suppression of antigen presentation by type 1-T helper cells Th1 [28]. IL-10 and interferon IFN-γ are required for the development of T regulatory cells Tregs, where ARG1 and CD40 play a role in this process. MDSC can alter the production of cytokines. Mice with tumors have decreased IL-7 and STAT5 signaling, which is important for B-cell differentiation, resulting in decreased circulating IgG levels. The population of tumor-associated macrophages (TAMs) promotes tumor progression. M-MDSC-derived macrophages retained most of the properties of their predecessors, including immunosuppressive function [29]. In hypoxic regions of solid tumors, M-MDSCs rapidly convert to TAMs and MDSCs also communicate with macrophages to enhance the protumoral activity of TAMs [30].

STAT3 is a repressor of anti-tumor immunity, and its expression impairs antigen presentation and inhibits the production of immunostimulatory cytokines, while promoting the expression of immunosuppressive molecules. This factor is present in most cancers and induces the production of inflammatory cytokines and growth factors such as IL-6, IL-10, IL-23, LIF, VEGF, and HGF [31,32,33]. When STAT3 activation is induced in myeloid precursors, this factor controls cell survival, transcription of immunosuppressive enzymes (ARG1 and iNOS), prevents myeloid-cell maturation and results in aberrant differentiation into immature MDSCs [34]. Some of the key regulators of MDSC accumulation and activity are the transcription factors STAT3 and NF-κβ. STAT3 enhances MDSC accumulation through several pathways. STAT3 and STAT5 inhibit IRF8, a crucial transcription factor that drives normal myeloid differentiation into monocytes and dendritic cells, and down-regulates the differentiation of MDSCs, when this is necessary to inhibit their pathological expansion [35]. The proinflammatory damage-associated molecular pattern DAMP is commonly found in the TME and activates MDSC through NF-κβ.

**Figure 1:**
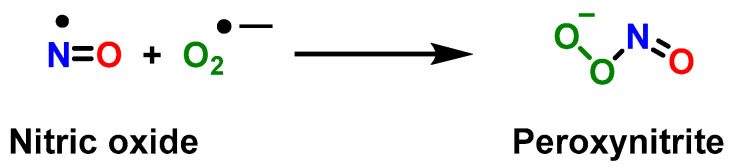
Reaction between the radical ^•^NO and superoxide anion ^•^O_2_^−^, yielding peroxynitrite.

STAT3 upregulates p47^phox^ and gp^91^, which increases ^•^NO and peroxynitrite [1]. Peroxynitrite is formed when nitric oxide ^•^NO reacts with superoxide anion ^•^O_2_^−^ [1] due to the overexpression of two subunits of NADPH oxidase, p47^phox^ and gp^91^ (derived from phosphorylation of STAT3, a hallmark of MDSC) [32]. Peroxynitrite is an anion derived from the reaction of ^•^NO with ^•^O_2_^−^, Figure 1.

Peroxynitrite is unstable and breaks down into ^•^NO_2_ and ^•^OH. ^•^NO_2_ reacts with the tyrosine residues of key immune cell signaling proteins and inactivates them by nitration. Nitration alters the TcR and MHC (major histocompatibility complex) on antigen-presenting cells, which prevents T cells from recognizing antigens [36]. Therefore, targeting MDSCs with peroxynitrite inhibitors is a therapeutic pathway to improve the response to immunotherapy [37]. The two MDSC subsets use different mechanisms to suppress T-cell proliferation. The PMN-MDSC expresses high levels of ROS and low levels of ^•^NO, and the M-MDSC expresses low levels of ROS and high levels of ^•^NO. Arginase 1 expression is common to both [38] and suppresses antigen-specific T-cell proliferation to an equal extent despite having different mechanisms of action [39].

## 3. Role of Myeloid-Derived Suppressor Cells in Cancer

In the early 1970s, initial research was published linking tumor growth to the proliferation of immunosuppressive myeloid cells. Research conducted in the 1980s and 1990s by Diana Lopez, Jim Talmadge, M. Rita Young, and Hans Schreiber showed that different types of myeloid cells suppressed immunological function in tumor cell growth [3].

In most types of cancer, PMN-MDSCs account for more than 80% of MDSCs. There is another small group (less than 3%) of cells with myeloid colony-forming activity that represent a mixture of myeloid progenitors and precursors [40].

Myeloid-derived suppressor cells are present in virtually all cancer patients, impair adaptive and innate anti-tumor immunity, and promote tumor progression by non-immune mechanisms. Their widespread presence combined with their diverse peritumoral activities makes them a major obstacle to cancer immunotherapy [41]. MDSCs have been detected in cancer patients and mice with tumors for more than 30 years. They inhibit antitumor immunity and act through CBI-independent signaling pathways [42,43]. In addition, MDSCs interfere with antibody treatments and promote tumor development via a variety of non-immune pathways [44].

Tumor immunity represents a new avenue for improved cancer therapy. Evasion of the immune system is a key feature of tumors [45]. To successfully establish themselves in a host and continue to grow, tumor cells use biochemical signals to hide from the host’s immune response and remain undetected. Immunotherapy aims to restore the immune response and immunity to cancer and has revolutionized cancer therapy in recent years. However, immunosuppressive rogue cells such as tumor-associated macrophages TAM, tumor-associated neutrophils TAN, regulatory T cells Treg, regulatory dendritic cells RegDC, cancer-associated fibroblasts, and MDSCs remain a major obstacle to immunotherapy and contribute to treatment failure, reduced life expectancy, and poor prognosis [46,47,48]. Checkpoint blockade immunotherapy CBI has been a revolution in cancer treatment because the patient’s adaptive immune system can eradicate malignant cells once the immunosuppressive mechanisms are neutralized [43]. Immune checkpoint inhibitors have successfully improved outcomes in various tumor types, and immune cell-based therapy is also gaining attention [49]. However, this IBC treatment is only effective in a certain group of cancer patients, as other immunosuppressive mechanisms appear to block T-cell-induced anti-tumor immunity [50].

As seen, immunosuppression plays a crucial role in tumor progression and contributes to the frequent failure of immunotherapy treatments and potential cancer vaccines, so it is necessary to address the study of the inhibition of these MDSCs to ensure the viability of the cancer immunotherapy approach. Elimination of suppressor factors is now recognized as a necessary step toward effective cancer immunotherapy. Drugs such as gemcitabine can be used to completely remove MDSCs from the body. There was no appreciable decrease in the number of B and T cells, suggesting that this effect occurs only in MDSCs. In contrast, a study of 17 patients with early-stage breast cancer found that chemotherapy with doxorubicin and cyclophosphamide resulted in an increase in the number of MDSCs in the peripheral blood [51].

## 4. Role of Myeloid-Derived Suppressor Cells in COVID-19

MDSCs have been described in a number of viral diseases, including respiratory infections [52]. It is still unclear how these cells contribute to the development of infectious diseases. On the one hand, MDSCs hinder the body’s ability to eliminate pathogens from the bloodstream and from the site of infection by suppressing the actions of effector-immune cells. On the other hand, MDSCs can prevent host organs from suffering lethal dysfunction by limiting the hyperinflammation and “cytokine storm” caused by infection [53].

During the infective process of COVD-19, pathogen-associated molecular patterns PAMPs from the replication of SARS-CoV-2 in host cells are recognized by a variety of membrane PRR pattern recognition receptors, including Toll-like receptors TLR-3, -4, -7, and -8; in addition to the porin domain of the NOD-like receptor family NLRP3, the retinoic acid-inducible RIG1, melanoma differentiation-associated protein 5 MDA5, and LGP2 are also present. Single-stranded RNA of SARS-CoV-2 is recognized by TLR-7 and -8, while double-stranded RNA intermediates are bound to TLR -3, RIG1, LGP2, and MDA5. SARS-CoV-2 proteins are recognized by TLR-4 and NLRP3 [54,55,56].

Moreover, viral replication triggers the synthesis of host-specific threat-associated molecular patterns DAMPs, which are then secreted extracellularly by injured or dying infected cells after rupture of the plasma membrane and recognized by cells bearing pattern recognition receptors PRRs. Calprotectin S100A8/A9, HMGB1 protein, mitochondrial DNA mt-DNA, and extracellular secreted nicotinamide phosphoribosyl transferase eNAMPT are important DAMPs associated with COVID-19 [57,58,59]. In response to PAMPs and DAMPs, many cell types secrete inflammatory mediators, chemokines, and growth factors such as interleukins IL-1B, IL-6, IFNα/β, TNF-α, chemokine ligand CXCL8/IL-8, CXCL10, chemokine ligand CCL5, granulocyte colony-stimulating factors G-CSF, and granulocyte-macrophage GM-CSF [60,61].

Recent studies point to a link between tissue damage and inflammation, in which the damage-associated molecular patterns of DAMPs play a key role in the etiology of severe COVID-19 [62]. NETs (neutrophil extracellular traps) are involved in the pathogenesis of COVID-19 and this can be seen in the fact that treatment of healthy neutrophils with serum from COVID-19 patients triggers NET release; and, in general, SARS-CoV-2 stimulates neutrophils to release NETs [63]. Several components of NETs, together with factors such as oxidative stress, contribute to the release of endogenous DAMPs, leading to severe hypoxia and ultimately acute respiratory distress syndrome ARDS in patients with severe COVID-19 [64].

The innate immune sets the adaptive response, which begins its activation within days [65]. Antigen-specific B cells, CD4+ T helper cells, and CD8+ cytotoxic T cells work together to orchestrate the adaptive response. However, CD4+ T helper cells are more prevalent than CD8+ cytotoxic T cells in SARS-CoV-2 [66] The sequencing of the immune response implies that innate immunity is activated first, followed by adaptive immunity, acting synchronously to eliminate the virus and damaged cells [67,68]. After the pathogen is eliminated, a series of immunoregulatory cell populations terminate the inflammatory response and restore tissue homeostasis. There is a multifactorial risk that can affect the course of COVID-19 disease, including hypertension, cancer, diabetes, as well as respiratory, cerebrovascular, and chronic kidney diseases [69]. These comorbidities are associated with an immunocompromised state characterized by an impaired immune response and decreased ability to fight viruses, as well as advanced age [70,71].

Myeloid cells play an important role in the pathogenesis of SARS-CoV-2, as evidenced by the frequent observation of a huge expansion of the myeloid-cell compartment and a decrease in the leukocyte compartment [72,73]. In COVID-19, myeloid cells are characterized by decreased antigen presentation and increased immunosuppressive characteristics, both of which are consistent with the profile of MDSC [73]. M2 macrophages, regulatory dendritic cells, regulatory T cells, and myeloid-derived suppressor cells are the primary immunoregulatory cell subsets that contribute to the attenuation of inflammation [53,74,75,76].

Although MDSCs can suppress a range of immune cells (including NK and B cells), their main goal is to induce T-cell immunosuppression. Therefore, evaluating their ability to block immune effector-cell activity is a critical component of understanding MDSCs [53,77,78]. In patients with COVID-19, MDSCs have been studied both in the peripheral blood and, more specifically, in the airways. As noted by Dean et al. 2021, large numbers of Arg1-expressing PMN-MDSCs were found in the lungs of COVID-19-deceased patients. This finding suggests that SARS-CoV-2 infection begins in the upper airways but progresses to the lower airways, where local recruitment of MDSCs is observed [79]. L-arginine is converted to ornithine via the enzyme arginase 1 Arg1, which inhibits T-cell proliferation and causes significant molecular changes in T cells, such as low CD3ζ chain expression and reduced IFN-γ production [80]. Interleukin IL -10 and transforming growth factor TGF-β produced by MDSCs inhibit T-cell activation and recruit regulatory Treg cells, respectively [81]. In addition, MDSCs can bind to PD1 molecules on T cells via their ligand PDL1 and induce T-cell death via cell-to-cell interactions [82].

PMN-MDSC generate ROS and nitric oxide ^•^NO, and this allows the formation of peroxynitrite, which in turn is broken down to generate two new radicals, ^•^NO_2_ and ^•^OH, with a high oxidative capacity [83]. ^•^NO_2_ induces T-cell receptor nitration on CD8+ cells (they are cytotoxic, like CD4+ T-helper cells, and express the TCR-cell receptor), during cell-to-cell contacts [84]. This nitration causes T cells to lose their ability to bind to the phosphorylated MHC (major histocompatibility complex) and therefore are unable to perform their function and respond to specific antigens, resulting in antigen-specific T cell tolerance [85].

Finally, according to a number of studies, there is a correlation between the number of MDSCs and the severity of COVID-19. Patients who required treatment in the ICU had more PMN-MDSCs than patients who did not, according to a 2020 study by Sacchi et al. [86]. In addition, Reizine et al. 2021 discovered that patients with acute respiratory distress syndrome ARDS have more PMN-MDSCs and M-MDSCs than individuals with moderate disease [87].

## 5. Reactive Oxygen Species and Its Generation

In cell chemistry, reactive oxygen species ROS are highly reactive species formed from the diatomic oxygen O_2_, examples of ROS being superoxide anion, hydrogen peroxide, hydroxyl radical, singlet oxygen, and alpha oxygen. In a biological and molecular context, ROS species are by-products of normal oxygen metabolism and play an important role in cell signaling and homeostasis, and on a physiological level they are intrinsic to cellular functioning (at low and stationary levels in normal cells [88]).

**Figure 2:**
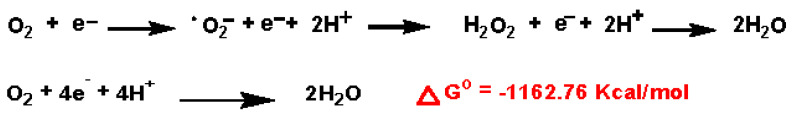
O_2_ reduction chain to ^•^O_2_^−^, H_2_O_2_ and H_2_O.

Chemically, the ^•^O_2_^−^ anion is a by-product of the respiration chain in the inner-mitochondrial membrane [89], shown in Figure 2. As a necessary condition in processes such as chemical reactions, the Gibbs free energy determines how much work a thermodynamically closed system can do at a given temperature and pressure. Since the Gibbs energy for the sequential reduction of O_2_ with H^+^ and e^−^ is negative Figure 2, the reaction proceeds spontaneously (∆Go ≤ 0) [90].

In the next ROS step, ^•^O_2_^−^ is converted to hydrogen peroxide (H_2_O_2_) by the superoxide dismutase family of enzymes [91], and in the presence of the enzyme myeloperoxidase MPO and chloride ion, generates hypochlorous acid HOCl [92], a potent antibacterial agent that is part of the innate immune system’s antimicrobial arsenal. Other potent oxidants involved are peroxynitrite ONOO^−^ (formed by the reaction between the superoxide anion and the nitric oxide ^•^NO) and hydroxyl radical (^•^OH, formed by the Fenton or Haber–Weiss reaction or by the decomposition of ONOO^−^, ^•^NO_2_ and ^•^OH).

In antimicrobial responses, mitochondrial and NOX enzymes are the most important and best-studied ROS generators in macrophages. However, macrophages have other ROS sources. The oxidation of hypoxanthine and xanthine to uric acid is catalyzed by the enzyme xanthine oxidase (XO), which degrades xanthine and is predominantly localized in the cytosol, where it plays a crucial role in purine nucleotide catabolism [93]. Hydrogen peroxide is generated as a by-product during oxidation. XO has important physiological functions; a few studies have examined its activity in macrophages and suggest that it may be involved in inflammasome activation and cytokine expression [94].

NADPH oxidase, the enzyme complex known as NOX (nicotinamide adenine dinucleotide phosphate oxidase), is bound to the cell membrane in the extracellular space and is also found in the membranes of phagosomes used by neutrophils to engulf bacteria [95]. Human isoforms of the catalytic enzyme complex include NOX1–5 and DUOX1–2. The first mammalian NOX to be discovered was NOX2, the phagocyte NADPH oxidase [96]. NOX1 is extensively expressed in different cells, particularly in colonic epithelial cells [97] and NOX2 is expressed mostly in phagocytes, with lower manifestation in vascular smooth-muscle cells and human endothelial cells [98,99].

NOX catalyzes the production of a superoxide free radical by transferring one electron to oxygen from NADPH. Neutrophilic NOX produces ^•^O_2_^−^ almost instantaneously, whereas the vascular enzyme produces ^•^O_2_^−^ in minutes to hours, and in this case the radical anion appears to be released mainly intracellularly.

In humans, a lack of ROS triggers chronic bacterial infections, while its uncontrolled release causes pathologies due to excessive inflammation [100]. Professional phagocytes such as neutrophils (polymorphonuclear neutrophils PMNs), eosinophils, monocytes, and macrophages use superoxide-generating NADPH oxidase NOX as part of their arsenal of antimicrobial pathways to generate high levels of ROS [101]. To generate superoxide, NADPH oxidase transfers an electron from NADPH to an oxygen molecule [102].

Peroxisomes are organelles found in the cytoplasm of almost all eukaryotic cells. Superoxide and hydrogen peroxide are byproducts of fatty-acid oxidation. Because peroxisomes can rapidly generate and scavenge H_2_O_2_ and ^•^O_2_^−^, they play a key role in controlling the ebb and flow of reactive oxygen species ROS.

Cyclooxygenases and Lipoxygenases; both families of COX and LOX generate ROS as a by-product, in the arachidonic acid AA-metabolizing process.

**Figure 3:**
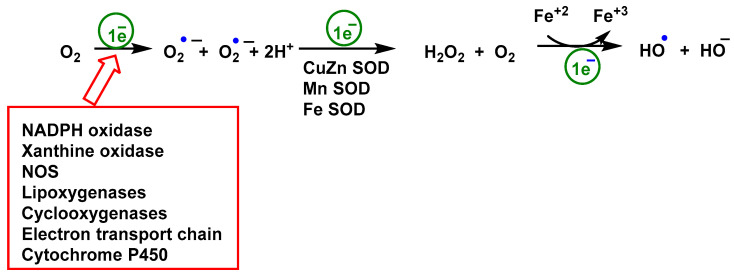
Chemical and enzymatic reactions generation of ROS. Reduction of O_2_ produces ^•^O_2_^−^, it is the precursor for all other ROS subspecies. Rapid dismutation of superoxide to peroxide allows its further conversion to OCl by myeloperoxidase MPO [92] or by Fe^3+^ to ^•^OOR or ^•^OH. The Fenton reaction, in which peroxide is oxidized by Fe^3+^ with ^•^OH as a reactive intermediate, rarely occurs in cells. O_2_ is rarely excited to ^1^O_2_ by radiation in mammals.

All three types of life forms, bacteria, archaea, and eukaryotes, have heme monooxygenases called cytochrome P450 CYP enzymes. These enzymes play an important role in the metabolism of sterols, fatty acids, eicosanoids, and vitamins, as well as in the detoxification of drugs and xenobiotics [103,104,105]. Chemical and enzymatic reactions generating superoxide ^•^O_2_^−^ are shown in Figure 3.

ROS can be divided into two families: (i) reactive free radicals, being the first formed by the superoxide anion ^•^O_2_^−^, the hydroxyl ^•^OH, the alkoxyl ^•^OOR and peroxyl ^•^OOH radicals [106]; and (ii) the non-radical species hydrogen peroxide H_2_O_2_ [91], singlet oxygen ^1^O_2_ [107], and ozone O_3_ [108]. The radical ^•^O_2_^−^ quickly dismutates to peroxide, either spontaneously in the presence of water or catalyzed by superoxide dismutase, SOD1, SOD2, and SOD3 [109]. Species ^•^O_2_^−^ and H_2_O_2_ are the two most abundant ROS species in cells, the first being more reactive than the second. Superoxide can only pass through cell membranes via ion channels, such as voltage-dependent anion channels VDACs [110] unlike H_2_O_2_, which is more freely diffusible [111]. An increase in ^•^O_2_^−^ levels is associated with oxidative stress and cellular damage because it can react with lipids, proteins, and DNA [83].

## 6. Role of Reactive Species on Innate and Adaptive Immunity

The immune system includes cells, organs, proteins, and tissues throughout the body and consists of components such as leukocytes, spleen, bone marrow, lymphatic system, thymus, tonsils, adenoids, and appendix. There are three types of immunity in humans: innate, adaptive, and passive.

**Figure 4:**
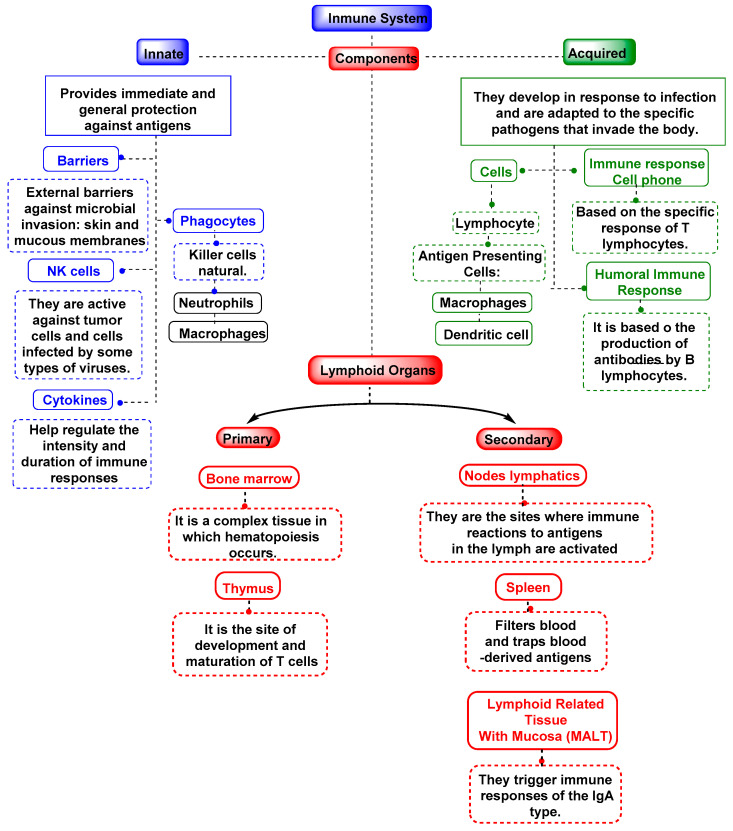
Mechanisms of the immune system, both innate and adaptive.

While innate immunity and acquired immunity were once thought to be unrelated, recent research has shown that the two are in fact closely intertwined [112]. All multicellular organisms have an innate immune system, a rudimentary defense mechanism that relies on macrophages, neutrophils, the complement system, natural killer cells, gamma delta T lymphocytes, and dendritic cells to function [113]. Figure 4 is an illustration of the immune system.

As the name suggests, innate immunity is something that every person is born with, and it immediately protects the body from some pathogens. The skin and the mucous membranes of the throat and intestines are part of this innate immunity, which serves as the first line of protection against infectious agents. All plants, fungi, mammals, and even the earliest multicellular organisms have innate immunity, a form of nonspecific immunity that has evolved over millions of years. Immune responses to pathogens are complicated by ROS in innate immunological processes such as the respiratory burst and inflammasome activation. During a respiratory burst, many cell types rapidly release oxygen and hydrogen peroxide. Phagocytic macrophages and neutrophils are myeloid cells that play a key role in the respiratory burst, which is necessary for the subsequent destruction of bacteria or other pathogens that have been internalized. For a respiratory burst to occur, NADPH oxidase activity must be increased by a factor between 10–20, which increases oxygen demand [114].

Peroxynitrite levels increase when ^•^NO and ^•^O_2_^−^ are present; these levels contribute to the “cauldron effect” when the environment is acidic. Pathogen lysis is facilitated by this mechanism, which is activated during phagocytosis due to the acidic environment of the phagosomes [115]. The so-called “cauldron effect” in the phagosome provides the cell with an isolated environment in which it can carry out the destruction of foreign bodies. Here ROS, ^•^NO, and RNS work together to trigger redox [116]. Superoxide ^•^O_2_^−^ is generated on the membranes of the endosome of the phagocytosing cells, with the involvement of NADPH oxidase NOX [117].

Nitric oxide ^•^NO is a cellular signaling molecule, found in vascular endothelium cells, platelets, macrophages, and neuronal cells. ^•^NO suppresses platelet aggregation, limits endothelial adhesion of neutrophils, defines basal vascular tone, and modulates myocardial tone. To maintain steady vascular tone, vascular endothelial cells secrete a steady stream of ^•^NO. The guanidine nitrogen atoms of L-terminal arginine are oxidized, producing ^•^NO [117].

With a half-life of only about 10^−2^ s, peroxynitrite ONOO^−^ is a potent oxidant with a short half-life. Lipid peroxidation, inactivation of enzymes and proteins, and mitochondrial dysfunction are just some of the effects of its derivatives. In cells such as macrophages, ONOO^−^ is critical for the destruction of invading pathogens [118]. Inadequate control of its production has been associated with increased risk of cardiovascular disease, neurological disorders, and cancer. The byproducts of peroxynitrite degradation are ^•^NO_2_, ^•^OH, and ^•^CO_3_^−^ [119].

Myeloperoxidase MPO, a hydrogen peroxide oxidoreductase, is found in macrophages as well as various other cell types, fluids (saliva, synovial fluid, and semen), and tissues (heart, kidney, skin, liver, and placenta) [92]. Most of the enzyme originates from neutrophils, where it is found in lysosomes [120]. MPO catalyzes the oxidation of Cl^−^ anion using H_2_O_2_ to produce HOCl, a highly reactive chlorinating and oxidizing agent and the primary strong oxidant produced in significant amounts by neutrophils [121].

Lymphocytes perform adaptive immunity by remembering the appearance of foreign substances and building a new immune response to protect the body from future infections. Certain immune cells and antibodies are required for adaptive immunity. T and B cells play a central role in the adaptive immune response, which aims to eradicate infections and create an immunological memory [122].

Uniquely, vertebrates have adaptive immunity, so they can recognize and destroy specific pathogens. This immune response can identify and remember pathogens, generating increasingly potent consecutive responses to the re-encountered pathogen. It consists of two parts: one called humoral, organized by B cells (they are responsible for producing antibodies), and the other called cellular, represented by T helper cells (they support the activity of other immune cells by releasing cytokines) [123].

High exposure to ROS harms the T-cell response. Adam J Case et al., 2011, demonstrated that a thymus-specific elevation of mitochondrial ^•^O_2_^−^ disrupts normal T-cell development and impairs the function of the mammalian adaptive immune system. Conditional loss of SOD2 increased ^•^O_2_^−^, apoptosis, and developmental defects in the T cell population, resulting in immunodeficiency and susceptibility to the influenza A H1N1 virus. This phenotype was rescued with mitochondrial targeted ^•^O_2_^−^ -scavenging drugs [124].

Activation and proliferation of B cells rapidly induces the production of ROS, which is why they are equipped with robust antioxidant systems, otherwise they require exogenous antioxidant supply [125]. NOX2 produce ^•^O_2_^−^ during the early B-cell activation, and mitochondrial respiration can generate ^•^O_2_^−^ and ROS for later stages of B-cell activation [126].

## 7. Role of Reactive Oxygen Species in Cancer

MDSCs (as well as producing ROS themselves) arise in environments that are undergoing oxidative stress, such as tumors, which implies a close relationship between MDSCs and ROS [84]. A major source of ROS is found in the mitochondria, during oxidative phosphorylation OXPHOS, a metabolic process that uses energy released by nutrient oxidation to produce adenosine triphosphate ATP. An alternative mechanism is glycolysis, which oxidizes glucose to obtain energy for the cell, and consists of 10 consecutive enzymatic reactions that convert glucose into two pyruvate molecules.

In contrast to the inefficient energy production in glycolysis and the PPP pathway, the efficiency of energy production in the mitochondrial respiratory chain is much higher. The constant loss of electrons to O_2_ during mitochondrial ATP production is a disadvantage of aerobic respiration. The superoxide anion ^•^O_2_^−^ is formed from only 1–5% of the absorbed O_2_. Superoxide dismutase manganese Mn-SOD is an enzyme that neutralizes this free radical by converting it to hydrogen peroxide and water [127].

An advantage of glycolysis over OXPHOS lies in its better maintenance of redox balance, as it does not generate ROS on its own. Lian et al., 2017, observed that MDSCs counteract ROS from OXPHOS by up-regulating glycolysis, thus protecting MDSCs from apoptosis [128]. In addition, the self-release of ROS by MDSCs is one of the main mechanisms used to suppress T cells in humans. Nagaraj et al., 2007, demonstrated that ROS and peroxynitrite derived from MDSCs modify TCR and CD8 molecules. Through these modifications, CD8+ T cells lose their ability to bind to the phosphorylated major histocompatibility complex MHC and induce CD8+ T cell tolerance [85].

MDSCs suppress T cells and induce the regulatory T-cells (Tregs) expansion in cancer and in inflammation [129]. Treg are less susceptible to ROS-induced apoptosis compared to other T-cell populations [130], by a mechanism involving a greater secretion of thioredoxin or hemeoxygenase 1 (redox proteins) [131]. MDSCs also negatively regulate B-cell-mediated immune responses (and the production of antibodies) using ROS [132] together with ARG1 and ^•^NO [133].

Elevated rates of ROS can be detected in almost all cancers, where they promote many aspects of tumor development and progression. However, neoplastic cells also express increased levels of antioxidant proteins to detoxify ROS, suggesting a delicate balance of intracellular redox, thus ROS is required for cancer-cell function [134].

In cancer, ROS play a dual role: on the one hand, they can promote molecular genetic alterations that cause tumor initiation, growth and progression, as well as resistance to tumor treatment, but on the other hand, high levels of ROS have cytotoxic effects, inducing apoptosis or inhibiting resistance to anticancer treatments [135,136].

Cytokines and growth factors stimulate ROS production, for example, an increase in H_2_O_2_ and nitrite oxide levels was detected in tumor cells in response to IFN-γ and TNF-α. Dependent on the cellular location, elevated intracellular superoxide levels are produced through extracellular NADPH oxidase NOX or inner membrane mitochondria [137].

Many tumors result from sites with chronic oxidation, irritation, infection, or inflammation, and recent data have extended the idea that this concept is a critical factor of cancer development and progression [138].

Studies conducted over the past three decades have shed light on the process by which chronic oxidative stress can lead to chronic inflammation, which in turn can promote diseases such as cancer, diabetes, cardiovascular, neuro-logical, and pulmonary disorders. The transcription factors NF-κβ, AP-1, p53, HIF-1α, PPAR-γ, β-catenin/Wnt, and Nrf2 are all activated in response to oxidative stress [139]. The expression of inflammatory cytokines, chemokines, cell cycle regulatory molecules, and anti-inflammatory chemicals are all controlled by genes whose expression can be triggered by activation of these transcription factors.

Infections by bacteria, viruses, fungi, protozoa, and parasites; exposure to hazardous chemicals and radiation; inhalation of allergens; autoimmune or chronic disorders; obesity; use of alcohol or cigarettes; and a high-calorie diet are only some of the many factors that can lead to inflammation [140]. All of these causes have in common that the longer inflammation lasts, the higher the risk of cancer [141].

Acute inflammation is an initial stage marked by the triggering of innate immunity, should persist for only a short period of time, and is usually beneficial to the host. If inflammation lasts longer, chronic inflammation occurs, which can predispose the host to chronic diseases, such as cancer [142].

Inflamed cells produce soluble mediators, such as arachidonic acid metabolites, cytokines, and chemokines. They function by producing additional reactive species and recruiting myeloid cells to the site of injury, generating additional reactive species.

These key mediators can initiate signal-transduction cascades and alter transcription factors such as nuclear factor NF-κβ, signal transducer and transcriptional activator STAT3, hypoxia-inducible factor NF-κβ, AP-1 protein, and Nrf2 factor, all of which mediate immediate cellular stress responses [143].

The tumor microenvironment TME of many cancers is characterized by a high infiltration of monocytes, macrophages, dendritic cells, and granulocytes, and experimental and clinical studies show that most of the infiltrating myeloid cells remain in an immature state in the TME [144]. Additionally, tumor-infiltrating myeloid cells generate ^•^O_2_^−^, which can compromise the function and viability of adjacent cytotoxic lymphocytes (natural killer NK cells and T cells), oxidize DNA and trigger additional somatic mutations, and ultimately affect the redox balance in cancer cells, thereby controlling their proliferation and survival [145].

## 8. Role of Reactive Oxygen Species on COVID-19

A contagious infection known as coronavirus disease 2019 COVID-19 is caused by the severe acute respiratory syndrome 2 SARS-CoV-2 coronavirus. The first known case was detected in Wuhan, China, in December 2019, and the disease spread rapidly around the world. Currently, numerous scientific studies are investigating the effects of antioxidants, anti-inflammatory drugs, and natural immune boosters (e.g., vitamins C, E, D, N-acetylcysteine, melatonin, etc.) as adjuvant therapy to standard COVID-19 treatment. The role of reactive oxygen species in COVID-19 has been addressed in several reviews [146,147,148]. SARS-Cov-2 induces pro-inflammatory mediators such as the cytokines IL-1β, IL-6, IL-8, CXCL10, TNF-α and neutrophil recruitment occurs in response to the inflammatory process generated. The oxidative process is triggered in the respiratory tract by activation of the enzyme NADPH oxidase 2 NOX2 which generates the ^•^O_2_^−^ anion as part of its antimicrobial mechanism [149].

Severe COVID-19 is characterized by an impaired innate and adaptive immune response and by a massive expansion of MDSC cells. An increased ratio of MDSCs to memory CD8 effector T cells was observed in patients with severe ARDS due to COVID-19 compared to moderate COVID-19 patients, and the COVID-19 severity is correlated directly with lymphopenia and enlarged ARG1 activity [87]. There is a remarkable expansion of MDSCs, up to 90% of total circulating mononuclear cells in patients with severe disease, and up to 25% in patients with mild disease, with the frequency decreasing with recovery [150].

In the context of severe COVID-19 infection, neutrophilia and a high ratio of neutrophils to lymphocytes has been found to be a defining feature. ROS production was also found to be markedly elevated in COVID-19 patients, with mean values nine times higher than in healthy controls, and even much higher in mechanically ventilated patients [151]. ROS generation can be correlated with the neutrophil count, as has also been seen on many other occasions in patients with sepsis, unrelated to COVID-19. The increased neutrophil levels in patients with COVID-19 and during sepsis demonstrate a functional ability to form ROS, which contributes to the clinical features of acute illness and represents a potential new target for therapeutic intervention [152].

Tissue analyses and autopsies of deceased COVID-19 patients show neutrophilic infiltration of pulmonary capillaries, extravasation into alveolar spaces, and neutrophilic mucositis [153]. It has also been shown that there are more circulating extracellular neutrophil traps NET, indicating their activation [154].

In addition to neutrophil infiltration and ROS release, viral infections are associated with a decreased antioxidant defense. The transcription factor NRF2, which activates antioxidant defenses in pro-oxidant states, is usually translocated, but viral respiratory infections have been associated with the inhibition of NRF2-mediated pathways and activation of factor NF-κB, which promotes inflammation and oxidative damage during these infections [155]. Older COVID-19 patients also had lower levels of the antioxidant enzyme superoxide dismutase 3 SOD3, and this finding was related to disease severity [156]. Children are less susceptible to severe forms of COVID-19 because they have fewer reactive and adherent neutrophils without altered redox balance. Undoubtedly, the chain of events triggered by the oxidative stress state during SARS-CoV-2 infection contributes to the severity of the disease [157,158].

Therefore, neutrophilia can be considered to produce an excessive amount of ROS, which enhances the pathogenic response of the host immune system and exacerbates the disease [159]. Hypoxic respiratory failure, which occurs in the most severe cases of COVID-19, is caused by the deleterious effect of ROS on lung cell and red blood cell RBC activities [160,161,162].

Tonny Veenith et al., 2022, studied the production of ROS in 15 patients. They measured production by chemiluminescence detection on fresh blood samples and defined it as the LIT™ score. They compared the levels of LIT with hematological parameters such as total white blood cell WBC count and neutrophil count and found that the levels of LIT were strongly correlated with both. They examined the production profile of ROS in patients with COVID-19 and compared it with that of 12 ICU patients with sepsis who did not have COVID-19. The result of their study suggests that the levels of LIT were 45% higher in patients with sepsis compared with COVID-19 and that this may be related to the immature or dysfunctional phenotype of mature neutrophils observed in patients with severe COVID-19 disease. Neutrophils express ACE2, the viral spike protein receptor, and SARS-CoV-2 can directly stimulate NETosis and intracellular ROS production in neutrophils [152].

## 9. Stress in Endoplasmic Reticulum, a Major Driver of MDSC Influence of ROS

Rough endoplasmic reticulum RER and smooth endoplasmic reticulum SER are two subtypes of endoplasmic reticulum ER, which has important functions, such as protein folding. The ER performs cellular functions such as protein synthesis, folding, transport, and post-translational modifications, as well as lipid metabolism and calcium storage [163]. Alterations in protein folding lead to the accumulation of misfolded proteins in the lumen, resulting in ER stress, disrupting cell homeostasis. Unfolded protein response UPR triggers the production of ROS, which in turn promote ER stress [164].

ER stress has been reported to be a feature of MDSCs, especially in the case of PMN-MDSCs. The ER stress pathway is a major driver of MDSC, and it is activated by conditions in the TME, including low nutrient levels, inflammation, hypoxia [165], and intracellular accumulation of ROS [166]. In this context, excessive intracellular ROS accumulation can cause protein-folding stress by altering ER-resident calcium channels [167], generating by-products of lipid peroxidation, forming stable adducts with ER-resident protein chaperones [166]. The physiological function of the ER is closely linked to the cellular redox state, and recent studies suggest that ROS production is related to ER stress, acting as upstream signaling [168]. Other pathological factors such as hypoxia and inflammation can trigger ER stress. Conversely, ER stress induces inflammation and oxidative stress, maladaptive UPR induces apoptosis, and reduced interaction between the ER and other cell organelles can negatively affect normal physiological conditions [169]. Cellular ROS can directly induce oxidative modification of ER lumen proteins and then affect the function of ER molecular chaperones, resulting in the retention of folded proteins in the ER lumen and ultimately triggering ER stress [170]. Inhibiting ROS can inhibit the increase in ER stress and delay UPR activation, suggesting that ROS is the upstream signaling molecule that triggers the ER stress-mediated apoptosis pathway [171].

Endoplasmic reticulum is a more oxidative environment than the cytosol, mitochondria, or cell nucleus. The reduced/oxidized glutathione ratio GSH/GSSG appears to range from 1:1 to 3:1, very low related to the general ratio in a cell, usually 100:1 [172]. The incidence of excessive oxidative stress can modify protein folding and antioxidant supplementation could reduce UPR activation, oxidative stress, and apoptosis, and increases protein secretion in vitro and in vivo [173]. Recent findings indicate that ROS species are a signal generated by misfolded proteins in the ER, causing UPR activation and cell death. Intervention to reduce reactive oxygen species improves protein folding and cell survival and may provide a pathway to treat and/or prevent protein-misfolding diseases [173].

In tumor cells, the UPR signaling pathway serves as a stress-adaptation mechanism that supports their survival and propagation [174]. If ER stress is prolonged, the UPR triggers tumor cell apoptosis [175].

Endoplasmic reticulum stress can induce PMN-MDSC cells in hepatocellular carcinoma. Nan et al., 2017, investigated a series of HCC patients before anticancer treatment (*n* = 127), patients with mild active chronic hepatitis B (*n* = 10), liver cirrhosis due to hepatitis B (*n* = 10) and liver dysplastic node with hepatitis B (*n* = 10) in two center hospitals, and found that ROS production and activation of arginase I were the mechanism for PMN-MDSC-mediated immune suppression [176].

To maintain healthy ER homeostasis, cells have evolved an ER quality-control system ERQC. In mammalian cells, the ERQC system is responsible for protein secretion and proteostasis. It consists of endoplasmic reticulum-associated degradation ERAD, unfolded protein response UPR, and autophagy [177].

The degradation of misfolded proteins is critical to the ERAD system because ER stress results from an imbalance between protein-folding ability and demand, resulting in misfolded proteins in the cell [178]. Selective degradation of misfolded protein fragments is possible via the ubiquitin-proteasome system, UPS, and the autophagic-lysosomal system, ALS. When the number of misfolded proteins exceeds the destruction capacity of the ER, the UPR reaction is triggered, and the misfolded proteins that could not be digested by the proteasome are eliminated via autophagy [179].

Myeloid progenitor cells MP and lymphoid progenitor cells LP are multipotent stem cells that can give rise to a variety of immune cell types. The ERQC system has been shown in the literature to be critical for immune cell maturation and function [180].

As reported by Condamine et al. in 2014, the increased apoptosis and decreased survival of MDSCs in mice bearing a tumor were associated with the upregulated expression of the TRAIL receptor [181]. Subsequent studies by Liu et al., 2016 showed that the expression of TRAIL-Rs was upregulated and that the MDSCs exhibited higher levels of ER stress in tumor patients. LCL521, an acidic ceramidase inhibitor, caused MDSC death via a mechanism independent of apoptosis [182].

According to Tcyganov et al. 2021, IRE1α and ATF6 can regulate the suppressive effect of PMN-MDSC in malignant tumors because their deletion abrogates the suppressive activity of PMN-MDSC and slows tumor progression. IRE1α-XBP1 is the most widely used branch of the UPR (Unfolded Protein Response) [183].

Instead, IFN-γ signaling regulated the activity of M-MDSCs, suggesting that the UPR promotes immune evasion by controlling the survival and immunosuppressive capabilities of tumor MDSCs, which in turn is related to cancer progression. To control the enhanced suppressive activity of MDSCs triggered by the Toll-like receptor TLR-2, which promoted the differentiation of MDSCs into iNOS macrophages, IFN-γ produced by T cells was shown to be crucial [184].

ER stores calcium and maintains the balance between intracellular and extracellular Ca^2+^ concentrations, making it an important regulator of membrane transport and internal homeostasis. Calcium Ca^2+^ is a second messenger that controls various cellular processes, including proliferation, differentiation, motility, hormone production, glycogen metabolism, neuronal excitability, and death [185] Apoptosis can be induced in the early stages when the Ca2+ balance in the ER is disturbed [186]. Although the exact pathways are still being explored, generation of ROS and increased Ca^2+^ levels in ER are major events that may promote ER stress-mediated apoptosis [187].

## 10. Conclusions

Certain risk factors for severe COVID-19 disease, such as obesity and diabetes, are associated with oxidative stress. The SARS-CoV-2 virus leads to interactions that impair cellular metabolism and trigger oxidative stress responses. The resulting inflammation and oxidative stress can negatively impact the host. Similarly, cancer cells exhibit a sustained increase in intrinsic ROS generation that maintains the oncogenic phenotype and drives tumor progression. In cancer and COVID-19, MDSCs consist of the same two subtypes (PMN-MSDC and M-MDSC). MDSCs are a heterogeneous population of immature myeloid cells that proliferate in cancer, autoimmunity, and various chronic inflammatory diseases and impair the immunological function of effector T cells (and NK cells) by multiple mechanisms. In both cancer and COVID-19, their suppressive activity is mediated through the production of Arg1, IDO, TGF-β, and iNOS. Plasma levels of cytokines and other pro-inflammatory markers suggest that the expansion of these immature cells represents a systemic compensatory response aimed at reducing excessive inflammation.

Among the various subcellular components, the endoplasmic reticulum ER is one of the most unique and versatile parts, mainly because of the functions it performs. Under ER stress conditions, redox signaling plays a key role in the development of ROS, so there is growing interest in the link between ROS and ER stress in the pathogenesis of various diseases. ER homeostasis and ER stress have been described in detail in many reports. Although some ER regulatory agents or UPR components have shown potential for the prevention and treatment of certain diseases, there are still no clinical trials investigating these targeted agents for the treatment of diseases.

It depends on the stage and severity of the disease whether MDSCs are positive or negative during the SARS-CoV-2 infection process. If they appear late in the infection, when the immune system has overcome the virus, then their role is positive; otherwise, their appearance may be related to severe disease progression.

## Data Availability

Not applicable.
